# Effect of Highly Hydrophilic Superparamagnetic Iron Oxide Nanoparticles on Macrophage Function and Survival

**DOI:** 10.3390/jfb14100514

**Published:** 2023-10-12

**Authors:** Efterpi Korakaki, Yannis Vasileios Simos, Niki Karouta, Konstantinos Spyrou, Panagiota Zygouri, Dimitrios Panagiotis Gournis, Konstantinos Ioannis Tsamis, Haralambos Stamatis, Evangelia Dounousi, Patra Vezyraki, Dimitrios Peschos

**Affiliations:** 1 Laboratory of Physiology, Department of Medicine, School of Health Sciences, University of Ioannina, 45110 Ioannina, Greece; euterp.kor2@gmail.com (E.K.); ktsamis@uoi.gr (K.I.T.); pvezirak@uoi.gr (P.V.); dpeschos@uoi.gr (D.P.); 2 Nanomedicine and Nanobiotechnology Research Group, University of Ioannina, 45110 Ioannina, Greece; nikhkarouta@gmail.com (N.K.); pzygouri@gmail.com (P.Z.); dgourni@uoi.gr (D.P.G.); hstamati@uoi.gr (H.S.); edounous@uoi.gr (E.D.); 3 Department of Materials Science and Engineering, University of Ioannina, 45110 Ioannina, Greece; 4 Department of Biological Applications and Technologies, University of Ioannina, 45110 Ioannina, Greece; 5 Department of Nephrology, Faculty of Medicine, School of Health Sciences, University of Ioannina, 45110 Ioannina, Greece

**Keywords:** superparamagnetic iron oxide nanoparticles, macrophage, viability, reactive oxygen species, toxicity, immune response, inflammation

## Abstract

Superparamagnetic iron oxide nanoparticles (SPIONs) have garnered significant attention in the medical sector due to their exceptional superparamagnetic properties and reliable tracking capabilities. In this study, we investigated the immunotoxicity of SPIONs with a modified surface to enhance hydrophilicity and prevent aggregate formation. The synthesized SPIONs exhibited a remarkably small size (~4 nm) and underwent surface modification using a novel “haircut” reaction strategy. Experiments were conducted in vitro using a human monocytic cell line (THP-1). SPIONs induced dose-dependent toxicity to THP-1 cells, potentially by generating ROS and initiating the apoptotic pathway in the cells. Concentrations up to 10 μg/mL did not affect the expression of Nrf2, HO-1, NF-κB, or TLR-4 proteins. The results of the present study demonstrated that highly hydrophilic SPIONs were highly toxic to immune cells; however, they did not activate pathways of inflammation and immune response. Further investigation into the mechanisms of cytotoxicity is warranted to develop a synthetic approach for producing effective, highly hydrophilic SPIONs with little to no side effects.

## 1. Introduction

Superparamagnetic iron oxide nanoparticles (SPIONs) are a type of magnetic nanoparticle that contain iron oxide cores of multiple diameters (ranging between 4 and 100 nm) and a shell depending on the type of organic surfactant used during their synthesis [1]. SPIONs consist of nanoparticles of different magnetic phases, γ-Fe_2_O_3_ (maghemite) and Fe_3_O_4_ (magnetite). However, some other phases may be present, like α-Fe_2_O_3_ (hematite) [2] based on the synthetic method used in [3,4,5,6,7]. The magnetic cores of SPIONs allow them to be manipulated with an external magnetic field. The primary benefit of SPIONs is that, due to their small size, they can target specific areas of the body [8]. Thus, SPIONs currently have a variety of biomedical applications [9], such as magnetic resonance imaging (MRI) [10], drug delivery [11], and hyperthermia [12]. Furthermore, magnetic nanoparticles have recently been successfully employed in cancer therapy for applications such as photothermia or magneto-photothermia [13].

SPIONs, characterized by their exceptional magnetic properties, have gained widespread utilization in (the field of) MRI [14]. They have the potential to function as contrast agents in MRI, enabling the visualization of internal organs, tissues, and blood vessels [15]. SPIONs can also be utilized as drug delivery agents [16] (the organic shell of the magnetic nanoparticles offers a platform for attaching different drugs) and injected into the body, where they can be released in a more controlled way. Specific body areas can be targeted precisely by employing an external magnetic field, leading to more effective and targeted drug delivery [17]. Additionally, they can be used to track the movement of cells [18] or drugs within the body, increase the solubility of drugs, and facilitate easier administration [19]. Moreover, SPIONs have been employed in cancer treatment [20,21] and hyperthermia, which utilizes targeted heat to kill cancer cells [22]. By injecting SPIONs into the body and exposing them to an external magnetic field, they heat up and destroy cancer cells. Finally, SPIONs have also been explored in tissue engineering and regenerative medicine [23,24]. Their magnetic properties allow for manipulating cells and scaffolds in 3D [25], facilitating the creation of complex tissue constructs. On the whole, the medical sector benefits from the versatile applications of SPIONs [1], with ongoing research [26] ultimately leading to improved treatments [27] and therapies [28].

While SPIONs have found use in diverse biomedical contexts due to their distinctive magnetic characteristics, there is a prevailing concern about their potential toxicity, attributed to their size and composition [29,30] as well as the type of organic coatings [31]. Recent studies have shown that SPIONs can be toxic to cells in vitro [32], but the extent of the toxicity depends on the type and size of the SPIONs [33], as well as the cell type and organic shield. Furthermore, the toxicity of SPIONs is not well-defined, as different studies have reported varying levels of toxicity [34]. Some studies demonstrate that SPIONs can suppress inflammatory responses through mechanisms [35] or not develop immunotoxicity [36]. Research has shown that SPIONs can cause oxidative stress [37], inflammation [38], and other changes in the immune system [29]. Additionally, SPIONs can be taken up by cells and accumulate in the body, leading to adverse health effects [39].

Hence, it is imperative to conduct additional research to gain a deeper comprehension of the possible toxicity associated with SPIONs and the potential risks they pose to human health. Given their potential risks, it is of the utmost importance to investigate their immunotoxic effects and take appropriate safety measures when using them in biomedical applications. In this study, we researched the interaction between SPIONs, whose surface was properly modified to become highly hydrophilic, and a human monocytic cell line (THP-1), which is widely used to study inflammatory processes and immune responses. The aim was to gain insight into cellular uptake, cellular toxicity, and protein expression. 

## 2. Materials and Methods

### 2.1. Synthesis and Characterisation of SPIONs

**Figure 1:**
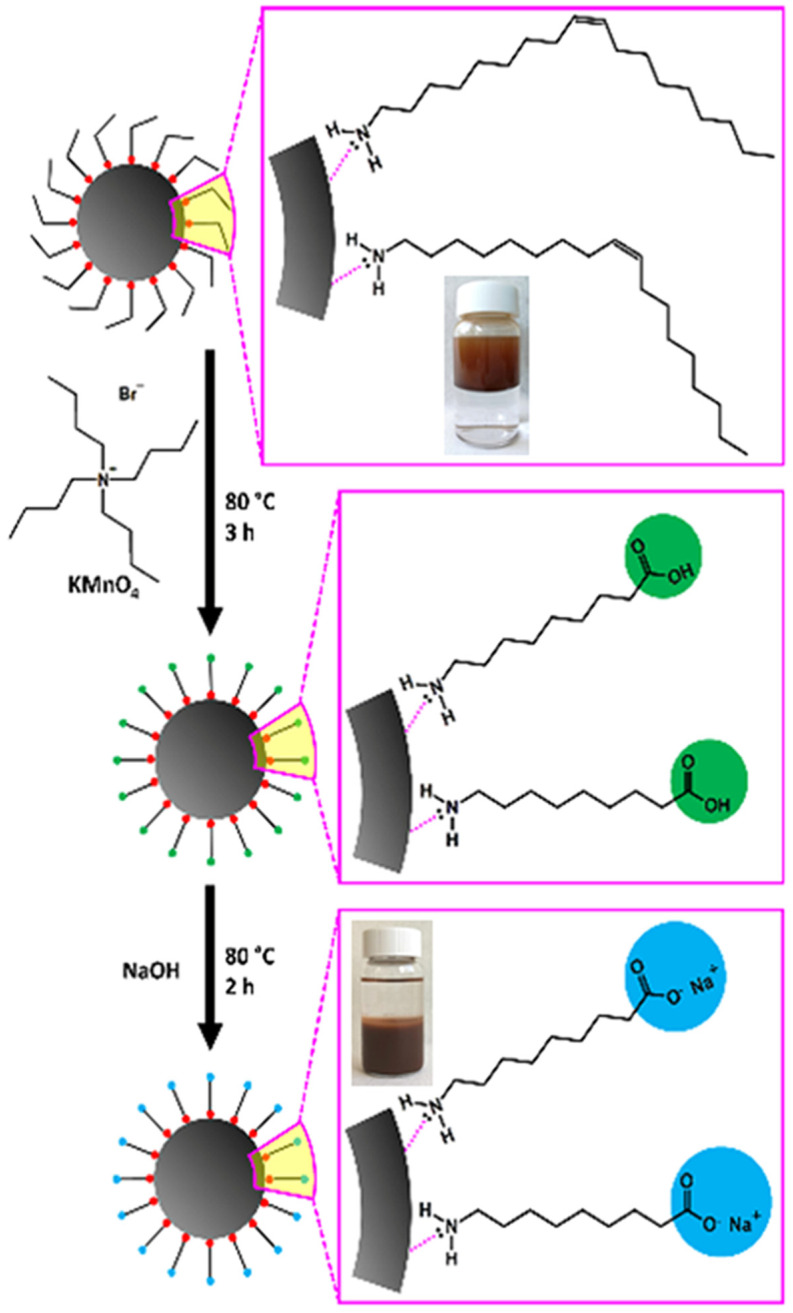
Schematic depiction of the “trimming” process of oleylamine. The inserted images display suspensions of o-SPIONs (2 mg/mL) in an organic solvent (n-hexane, **upper**) and h-SPIONs in distilled water (**lower**). Reprinted with permission from [40]. Copyright 2023 American Chemical Society.

Superparamagnetic iron oxide nanoparticles were produced through a novel modification procedure proposed in our previous work (Figure 1) [40]. In brief, 4 nm-sized SPIONs were initially synthesized through thermal decomposition. According to this reaction [41], the extremely small superparamagnetic cores of the SPIONs are coated with oleylamine, an organophilic molecule, precluding them from any utilization in biomedical applications. The “haircut” reaction was applied afterward on the oleylamine molecules, specifically on the carbon–carbon double bond located in the middle of the molecules’ length. The “trimmed” surface coating was carboxyl terminated, resulting in high hydrophilicity and great functionality. Physicochemical analyses and TEM images revealed that the core structure, diameter, and narrow size distribution of SPIONs remained unaffected by the modification reaction around 4 nm. The DLS characterization of aqueous dispersions of treated SPIONs showed a sharp monomodal size distribution of the h-SPIONs, and the polydispersity index (PDI) was found to be less than 0.25, indicating that the nanoparticles have a significantly narrow size distribution. In addition, TEM images of SPIONs after the “haircut” demonstrated improved dispersibility of the nanoparticles since the carboxyl groups on the surface increased the repulsive interactions between them. Mossbauer spectroscopy revealed the superparamagnetic nature of both samples while the magnetic properties remained unaffected. Furthermore, the aqueous dispersions of super hydrophilic SPIONs with pH values higher than 5 exhibited colloidal stability.

### 2.2. Cell Line and Antibodies

THP-1 cells are a human monocytic cell line derived from a patient with acute monocytic leukemia (TIB-202, ATCC, Manassas, VA, USA). These cells were used to study the immune response, inflammation, and other cellular processes. They are also useful for studying the role of transcription factors, cytokines, and other proteins in gene expression [42].

Primary rabbit mono-clonal antibodies heme-oxygenease-1 (HO-1) (1:750), Nuclear factor kappa-light-chain-enhancer of activated B cells (NF-κB) (1:1000), nuclear factor erythroid 2–related factor 2 (Nrf2) (1:750), Toll-like Receptor-4 (TLR-4) (1:1000), and secondary rabbit-specific horseradish peroxidase-conjugated antibody (1:1000) were obtained from Cell Signaling Technology, Inc. (Danvers, MA, USA). Primary mouse monoclonal antibody β-tubulin (1:500) and secondary mouse-specific horseradish peroxidase-conjugated antibodies (1:1000) were purchased from Santa Cruz Biotechnology (Santa Cruz, CA, USA).

### 2.3. Cell Culture

THP-1 were grown in RPMI-1640 medium (Sigma-Aldrich, St. Louis, MO, USA) supplemented with 10% heat-inactivated fetal bovine serum (BIOTECH, P40-37500 PAN), 1% Penicillin-Streptomycin (Biowest, L0022-020, Nuaille, Cholet, France), and 1% L-glutamine (Biowest, X0550-100, Nuaille, Cholet, France) in a humidified incubator with 5% carbon dioxide (CO_2_) and 95% air at 37 °C. 

### 2.4. Cell Viability Assay

To assess the cytotoxicity of SPIONs, we employed the 3-(3,4-dimethylthiazol-2-yl)-2,5-diphenyltetrazolium bromide (MTT) assay from Sigma-Aldrich Chemical Co., St. Louis, MO, USA. The MTT assay, a colorimetric method, measures cell viability by evaluating the ability of viable cells to convert a water-soluble yellow tetrazolium salt into an insoluble purple formazan compound. For our experiments, THP-1 cells (40 × 10^3^ cells per well) were seeded into 96-well plates, each with 100 μL of RPMI culture medium. Incubation was carried out at 37 °C with 5% CO_2_. In order to induce differentiation into macrophages, THP-1 cells were subjected to 4 mM phorbol-12-myristate-13-acetate (PMA) for one day. Following overnight incubation, we replaced the supernatant and introduced a fresh culture medium containing varying concentrations of SPIONs (0.1, 1, 5, 10, 20, 50, 100 μg/mL). After 24 and 48 h, we added 40 μL of MTT solution to the viable cells. The supernatant was subsequently aspirated after three hours, and 100 μL of dimethyl sulfoxide (DMSO) was added to each well. The quantification of viable cells was achieved by measuring absorbance at 540 nm and 690 nm using a microplate spectrophotometer (Infinite 200 Pro, Tecan, Switzerland). 

### 2.5. Detection of Apoptosis (Annexin-PI Assay)

The identification and quantification of apoptotic cells were conducted using the Annexin V-PI (Propidium iodide) assay using the manufacturer’s instructions (BioLegend Inc., San Diego, CA, USA). Briefly, differentiated THP-1 cells were incubated with 1, 5, and 10 µg/mL SPIONs for 24 h in 6-well plates (5 × 10^4^ cells per well). After the treatment, the cell supernatant was collected in Falcon tubes. Each well was rinsed with 2 mL of PBS buffer, which was then added to the corresponding tube. To detach the cells, 250 μL of trypsin-EDTA 1x (Biowest, Riverside, CA, USA) was added to each well, and the plate was incubated for 2 min. The wells were subsequently washed with 2 mL of PBS, and the resulting solution was added to the tubes. These tubes were centrifuged at 3000 rpm for 5 min, and the pellet was resuspended in Annexin V Binding buffer. Subsequently, 100 μL of the suspension containing 1 × 10^5^ cells was transferred into Eppendorf tubes. These tubes were then incubated with Annexin V-FITC and PI solution (BioLegend Inc., San Diego, CA, USA) for 15 min in the dark at room temperature. Following the incubation, the cells were promptly subjected to analysis using flow cytometry with the CyFlow Analyzer (Partec ML, Partec GmbH, Leipzig, Germany).

### 2.6. Cell Cycle Assay

A cell cycle assay was utilized to monitor cell progression through the various phases of the cell cycle, distinguishing cells within the G1, S, G2, and mitosis stages. The procedure involved staining cells with a fluorescent dye (PI) that binds to DNA, and subsequently measuring the fluorescence intensity within the cells. THP-1 cells (without PMA treatment) were subjected to exposure with SPIONs (at concentrations of 1, 5, and 10 µg/mL) for a duration of 24 h in 6-well plates (5 × 10^4^ cells per well). After removing the supernatant, each well was washed with 2 mL of PBS, and the collected material was transferred to Falcon tubes. The cells were trypsinized using 250 μL of trypsin solution and the plate was incubated for 2 min. Subsequently, the wells were again washed with 2 mL of PBS, and the resultant solution was added to the tubes. These tubes underwent centrifugation at 3000 rpm for 5 min, leading to the formation of a pellet. The pellet was then resuspended in 1 mL of ice-cold PBS and subjected to another round of centrifugation. The subsequent pellet was again resuspended, this time in 0.5 mL of ice-cold absolute ethanol. This was performed gradually, with ethanol being added drop by drop while gently vortexing. The tubes were subsequently frozen for a period of 7 days at −20 °C. Following this, they were centrifuged at 6000 rpm for 5 min, enabling removal of the supernatant. The cells were gently resuspended in 1 mL of PBS, and a mixture of 25 μL of 1 mg/mL PI and RNAase was added. Finally, the tubes were incubated in the dark for 30 min and then immediately subjected to analysis via flow cytometry (Partec ML, Partec GmbH, Leipzig, Germany).

### 2.7. Measurement of Intracellular Reactive Oxygen Species (ROS)

THP-1 cells were exposed to SPIONs (at concentrations of 1, 5, and 10 µg/mL) for a 24 h period in 6-well plates (3 × 10^5^ cells per well). The culture medium was collected into tubes, and the cells were subsequently washed with PBS. After incubating with trypsin and another wash with PBS, the previously collected medium was centrifuged at 3000 rpm for 5 min. This step generated a pellet, which was then resuspended in 2 mL of Hank’s Balanced Salt Solution (Biosera, Nuaille, France). The resuspended cells were transferred to Eppendorf tubes. Subsequently, fresh 2′,7′-dichlorofluorescin diacetate (DCFH-DA) with a purity of ≥ 97%, at a final concentration of 2.5 μM, was added to the tubes. The mixture was incubated for 30 min in the dark at 37 °C. Finally, 2 μM of propidium iodide (PI) was introduced to the solution. The tubes were then placed on ice, and the samples were immediately subjected to measurement using flow cytometry (Partec ML, Partec GmbH, Leipzig, Germany).

### 2.8. Western Blotting Analysis

Following treatment with SPIONs and Lipopolysaccharide (LPS), THP-1 cells underwent processing for protein expression analysis. In brief, cells were cultured with SPIONs (at concentrations of 0.1, 1, 5, and 10 µg/mL) for 24 h, as well as with 10 µg/mL LPS for 24 h, in 10 mL-volume Petri plates (4 × 10^6^ cells per well). The cells were then subjected to two ice-cold PBS washes, with the final wash (7 mL) being added to the dishes. Mechanical detachment of the cells was achieved by gently scraping them with a cell scraper on ice. The detached cells were collected in tubes and subsequently centrifuged (3000 rpm for 3 min) at room temperature, with the supernatant discarded. Each resulting pellet was resuspended in 1 mL ice-cold PBS on ice, followed by a centrifugation step (11,000 rpm for 8 s) at 4 °C. The pellets were then resuspended in 350 μL of ice-cold radioimmunoprecipitation (RIPA) buffer, supplemented with protease and phosphatase inhibitors from Sigma-Aldrich Chemical Co., St. Louis, MO, USA. The samples were kept on ice for 20 min, during which the first ten minutes involved gentle dispersion using a 21 G × 1 ½ needle syringe and vortex. Following this, the tubes were sonicated twice on ice-cold water for 20 s and then centrifuged (14,800 rpm for 20 min) at 4 °C. The resulting supernatants, constituting the total cell protein lysate, were collected in clean Eppendorf tubes. The protein content was quantified using the Pierce™ BCA Protein Assay Kit from Thermo Fisher Scientific Inc., Rockford, IL, USA. 

Equal amounts of protein were loaded onto a 10% sodium dodecyl sulfate-polyacrylamide gel (Sigma-Aldrich Chemical Co., St. Louis, MO, USA), and electrophoresis was conducted (30 min at 120 Volt, followed by 90 min at 180 Volt). The proteins from the gel were subsequently transferred to nitrocellulose membranes through electrophoretic transfer in ice (90 min at 180 Volt) at 4 °C. Each membrane underwent blocking with 5% non-fat milk in Tris-buffered saline (TBS) containing 1% Tween 20 (TBST 1x) overnight at 4 °C. Afterward, the membranes were rested on a rocker for 1 h at 25 °C, with a primary antibody diluted in 5% non-fat milk in 1x TBST buffer. The membranes were washed three times with 1x TBST buffer (5 min each), then incubated on a rocker under similar conditions with a secondary antibody. Following another round of washing, the membranes were developed (5 min) using enhanced chemiluminescence (ECL) substrate (Clarity Western ECL Substrate, Bio-Rad Laboratories Inc., Hercules, CA, USA). Finally, each protein band was captured using the Chemi-Doc™ MP Imaging System (Bio-Rad Laboratories, CA, USA), and subsequent analysis was performed using ImageLab (Bio-Rad Laboratories, CA, USA).

### 2.9. Statistical Analysis

All data were expressed as mean values +/− standard deviation (STDEV). Student *t*-test was used to determine the statistically significant difference between the mean values. A *p*-value < 0.05 was considered statistically significant. Results were analyzed using GraphPad Prism 8.0.1 software.

## 3. Results and Discussion

### 3.1. Cytotoxicity of SPIONs

**Figure 2:**
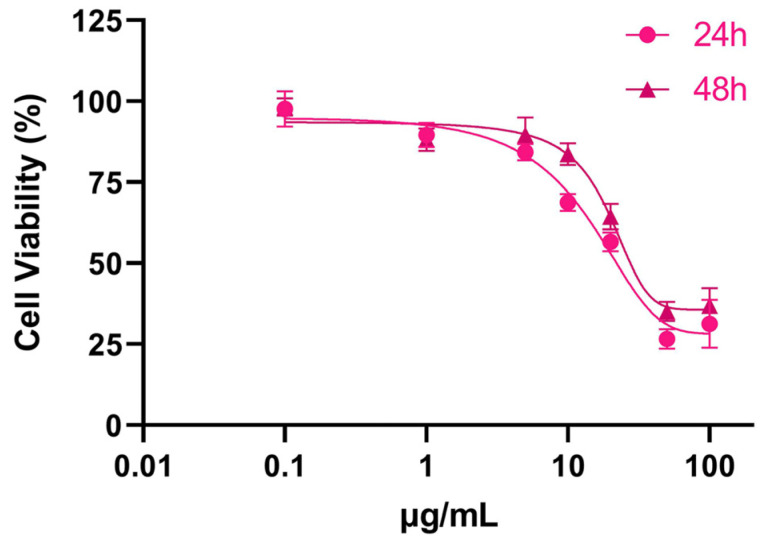
THP-1 cell viability after exposure to increasing concentration of SPIONs for 24 h and 48 h.

The MTT assay is a colorimetric method used to measure the metabolic activity of cells in a culture. It involves the addition of a colored compound called MTT to the cells. MTT is reduced by the enzymes present in the cells, resulting in the formation of an insoluble formazan product. The quantity of the formazan product produced corresponds directly to the cell count within the culture. SPIONs caused a dose-dependent decrease in THP-1 cell population. The cytotoxicity of SPIONs was primarily observed within the initial 24 h, as prolonging the exposure time did not affect THP-1 cell viability. The THP-1 cell population sharply declined at the doses of SPIONs from 5 μg/mL to 50 μg/mL (Figure 2).

Aiming to investigate how iron oxide nanoparticles impact the inflammatory reaction, namely cytokine release in response to TLR ligands, Wolf-Grosse et al. initially used human whole blood with 10 nm iron oxide nanoparticles (IONPs) to examine cell viability. The exposure of monocytes and granulocytes to 10 μg/mL IONPs for 6 h did not increase the number of dead cells [43]. Bare IONPs with a size range of 4.9–25.5 nm induced a mild reduction (~25%) in cellular viability of THP-1 macrophages after 48 h at 100 μg/mL [44]. Interpolymer complexed SPIONs modified with mannose, with a hydrodynamic size of ~305 nm (in media), were also non-toxic to M2 macrophages (differentiated THP-1 and RAW264.7 cells) at concentrations of 75–150 μg/mL for 10 h [45]. Potentially, the size and coating of nanoparticles are crucial factors to consider when studying their interactions with cells, and our highly hydrophilic SPIONs seem to be able to interact to a greater extent with THP-1 cells at low concentrations, negatively affecting their viability.

### 3.2. SPIONs-Induced ROS Generation

**Figure 3:**
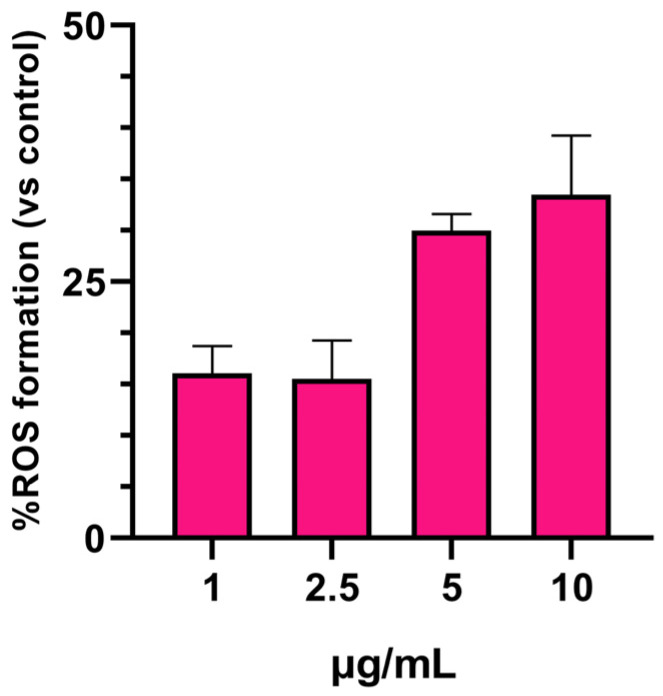
ROS formation after treatment of THP-1 cells with SPIONs for 24 h.

To assess the potential cytotoxicity of SPIONs, we conducted an ROS assay using flow cytometry. ROS were measured as highly reactive molecules capable of damaging cells and causing oxidative stress. The results showed that SPIONs induced a mild increase in ROS formation at doses higher than 2.5 μg/mL. After exposure to 5 and 10 μg/mL of SPIONs for 24 h, ROS formation remained similar, at approximately 30% (Figure 3).

The fact that iron oxide induces a rise in ROS production within cells via the Fenton reaction is widely acknowledged [46]. Nwasike et al. synthesized interpolymer complexed SPIONs (IPC-SPIOs) and studied their potential to scavenge ROS in macrophages, monocytes, and human umbilical vein endothelial cells (HUVEC) over 24 h using DCFDA [47]. The authors found that IPC-SPIOs did not generate ROS and demonstrated a dose-dependent ability to scavenge ROS. Specifically, ROS scavenging increased by ~50% in monocytes and ~75% in macrophages and HUVEC after 24 h of exposure to 150 μg/mL IPC-SPIOs. It is likely that the coating of IPC-SPIOs exhibits antioxidant activity. Nonetheless, the authors acknowledge the need for further investigation to fully understand the precise antioxidant or potentially oxidant potentials of IPC-SPIOs. Specifically, the relationship between the Fenton reaction, the IPC-SPIOs uptake, and decomplexation by the cells requires more exploration. In a previous study, we demonstrated that the SPIONs utilized in the present investigation exert varying effects on different cells. At a concentration of 10 μg/mL, these SPIONs increased reactive oxygen species (ROS) levels by approximately 1%, 32%, and 24% in HEK293, HeLa, and LMS cells, respectively [40]. In the current study, as mentioned earlier, the observed increase was approximately 30%.

### 3.3. SPION Influence on the Cell Cycle

**Figure 4:**
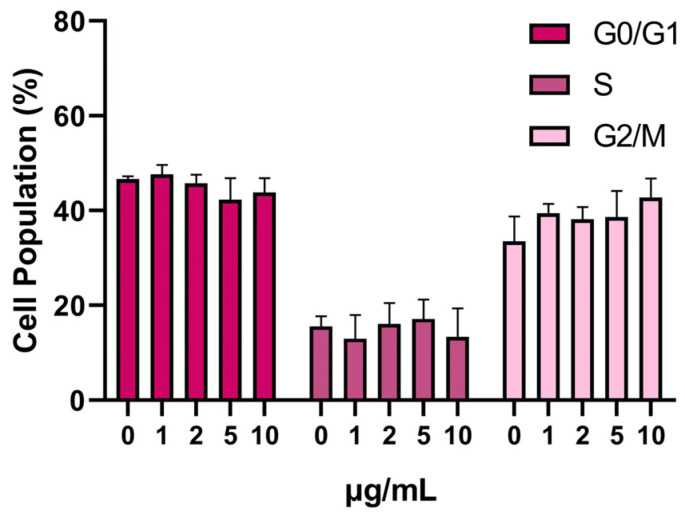
Cell-cycle analysis after treatment with SPIONs for 24 h.

The utilization of a cell cycle assay enabled the assessment of cell movement through distinct cycle phases, which include division, checkpoint identification, and the influence of SPION treatment. The differences between the control group and the samples exposed to our nanoparticles were assessed by analyzing the populations of cells in each phase. The analysis revealed a mild increase in mitosis (G2/M) after SPIONs treatment. Cells treated with 10 μg/mL of SPIONs showed an enhanced mitosis peak at around 43% of the total cell population, compared with 33% in the control cells (Figure 4).

Lafuente-Gomez et al. investigated the immunomodulatory effect of magnetic nanoparticles comprising a maghemite core (MNP) alone or with three distinct coatings (dextran, carboxymethyldextran, and dimer-captosuccinic acid) on THP-1 and RAW 264.7 cells. The authors concluded that coated MNP carrying 100 mg/mL ferum did not disrupt cell cycle progression [48]. In our study, we used significantly lower doses of SPIONs (up to 10 μg/mL), exerting minimal disruption to the cell cycle of THP-1 cells. At higher doses (20 μg/mL), the treatment interferes with the cell cycle, resulting in S-phase arrest for HEK293 and LMS cells, and G0/G1 arrest for HeLa cells [40].

### 3.4. Apoptosis

**Figure 5:**
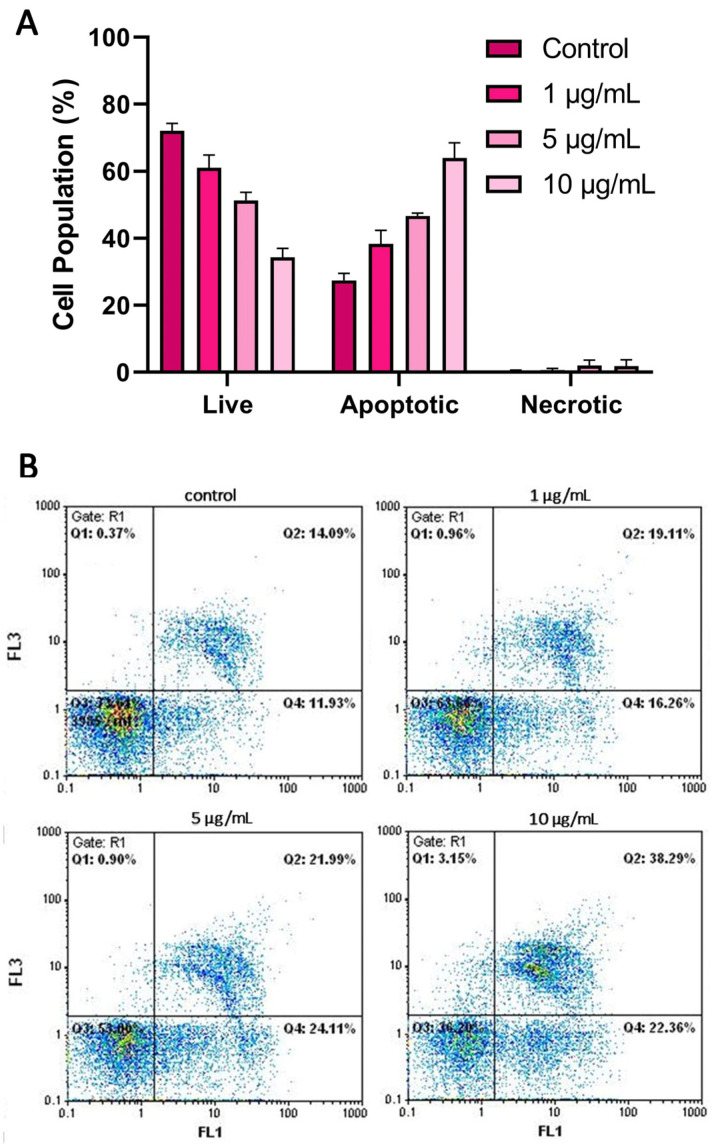
Induction of apoptosis/necrosis by SPIONs in THP-1 cells. Percentage of apoptotic cells after incubation with 1, 5, and 10 μg/mL SPIONS for 24 h (**A**). Representative flow cytometry images (Control: Live cells 73.61, Necrotic cells 0.37, Late Apoptotic 14.09, Early Apoptotic 11.93; 1 μg/mL: Live cells 63.67, Necrotic cells 0.96, Late Apoptotic 19.11, Early Apoptotic 16.26; 5 μg/mL: Live cells 53.00, Necrotic cells 0.90, Late Apoptotic 21.99, Early Apoptotic 24.11; 10 μg/mL: Live cells 36.20, Necrotic cells 3.15, Late Apoptotic 38.29, Early Apoptotic 22.36) (**B**).

For the apoptosis/necrosis analysis, cells were subjected to staining with Annexin V-FITC and PI, and their subsequent characterization into apoptotic, necrotic, or living categories was carried out using flow cytometry. The outcomes highlighted that SPIONs led to a gradual elevation in the population of apoptotic THP-1 cells, aligning with the administered dosage. Specifically, exposure to 1, 5, and 10 μg/mL for 24 h yielded a fold increase of 1.4×, 1.8×, and 2.4×, respectively, within the apoptotic cell subset (Figure 5).

The biocompatibility of SPIONs conjugated with cystine (Cy-SPIONs) was investigated using multiparametric flow cytometry assays on peripheral blood mononuclear cells (PBMCs) in dose-responses at 50, 100, and 200 μg/mL (for 24 h). The Cy-SPIONs had a similar size (3.95 nm) to those in our study. Interestingly, the apoptotic and necrotic population remained unaffected until the highest dose of 200 μg/mL [49]. Interestingly, a higher dose of SPIONs (20 μg/mL) did not induce apoptosis in HEK293, LMS, and HeLa cells after a 24-h incubation. However, extending the cells’ exposure to SPIONs (48 h) led to a significant increase in apoptotic cells, observed exclusively in LMS and HeLa cells. A critical factor determining the toxicity of SPIONs and the initiation of apoptosis seems to be the internalization of SPIONs within cells, resulting in ROS generation. This uptake was notably higher in HeLa and LMS cells compared with HEK293. Hence, the rapid uptake of SPIONs by THP-1 cells and the subsequent production of intracellular ROS, even at low doses of SPIONs (< 10 μg/mL), could potentially account for the reduced cell viability [40]. 

### 3.5. Western Blotting

In evaluating the biosafety of SPIONs, we aimed to investigate their impact on the immune system and inflammatory response, specifically focusing on the Nrf2/HO-1 and TLR-4/NF-κB pathways. Upon entering the human body, both SPIONs, and generally any nanoparticle, are identified as foreign substances. If SPIONs are recognized as “pathogens”, this recognition triggers the activation of TLRs, subsequently leading to the activation of both immune and non-immune cells and resulting in inflammation. TLR proteins play a pivotal role in initiating an innate immune response by activating the NF-κB-dependent pathway within inflammatory cells [50]. Notably, TLR4 enhances the expression of molecules involved in cell recruitment and proliferation, including cytokines and chemokines. This enhancement triggers the onset of an inflammatory response [51]. On the other hand, the transcription factor Nrf2 regulates multiple genes that confer protective activity within cells. For instance, it oversees HO-1, a stress protein that provides cytoprotection against oxidative stress [52]. Furthermore, Nrf2 demonstrates anti-inflammatory effects through its modulation of inflammatory cell recruitment [53], the enhancement of antioxidant enzyme production via regulation of the antioxidant response element (ARE) [52,53], and, ultimately, the inhibition of the NF-κB signalling pathway [54].

**Figure 6:**
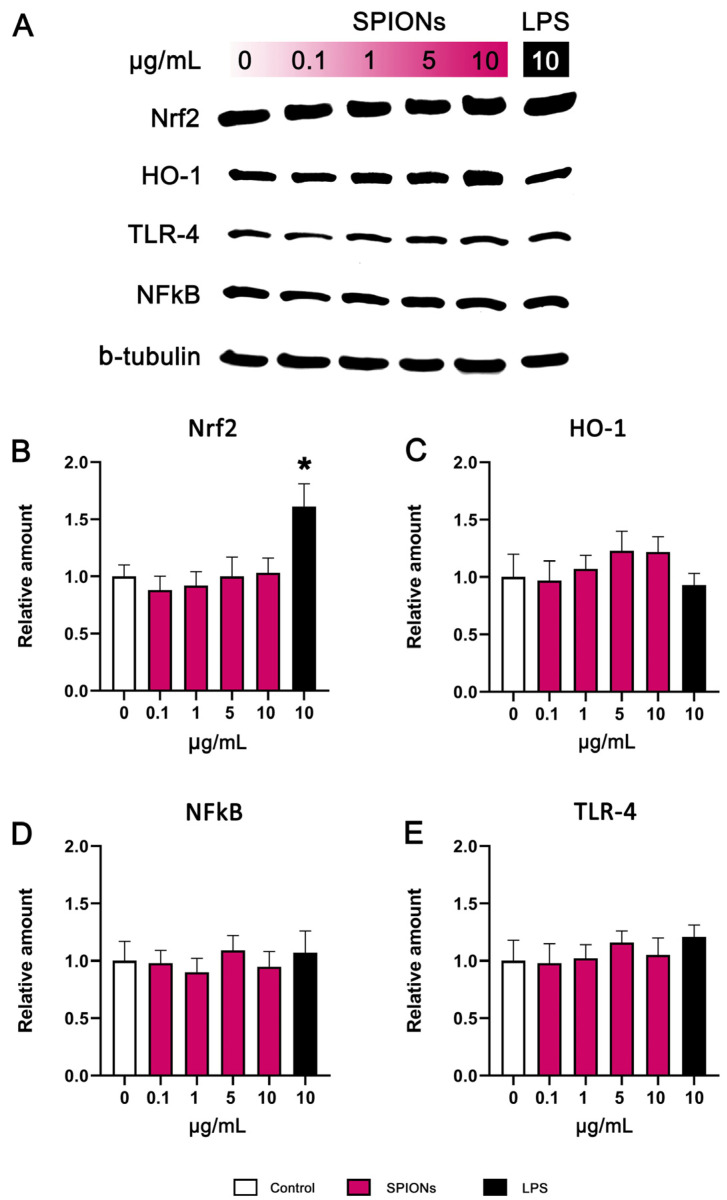
Representative Western Blot images of protein bands (**A**). Relative amount of Nrf2 (**B**), HO-1 (**C**), NfκB (p65) (**D**), and TLR-4 (**E**) in THP-1 cells treated with SPIONs (0.1, 1, 5, and 10 μg/mL) and LPS (10 μg/mL) for 24 h. b-tubulin was used as the loading control. *, statistically significant difference from control (*p* < 0.05).

Utilizing HO-1, NF-κB, Nrf2, TLR-4, and β-tubulin antibodies, we detected the specific proteins and measured their concentrations within our samples. As a positive control, we used 10 μg/mL of LPS. As shown in Figure 6, the expression of Nrf2 (Figure 6B), HO-1 (Figure 6C), Nf-κB (Figure 6D), and TLR-4 (Figure 6E) was not significantly altered by SPIONs in THP-1-derived macrophages. 

Data regarding the impact of SPIONs on molecular pathways that are correlated with inflammation and immune response are scarce. In 2016, Vogel et al. demonstrated for the first time that small-sized iron oxide nanoparticles (3 to 12 nm in diameter) at a concentration of 50 μg/mL for 6 h induce the activation of HO-1 and NAD(P)H:quinone oxidoreductase (NQO-1) through the Nrf2 pathway [55]. However, our analysis did not reveal any shifts in the expression of Nrf2 and HO-1. Research has shown that polyethylenimine-coated SPIONs at a concentration of 6.25 μg/mL induce macrophage activation in both THP-1 and RAW 264.7 cells through TLR-4 signaling [56]. These findings are consistent with Jin et al.’s study, where they demonstrated that exposure to clinically approved dextran-coated SPIONs, Resovist (ferucarbotran) and Feraheme (ferumoxytol), at doses of 50 μg/mL and 100 μg/mL for 18 h, stimulate macrophage autophagy and initiate an inflammatory response by activating the TLR-4 signaling pathway in RAW 264.7 cells [57]. In this case, we also did not record any changes in the TLR-4/NF-κB signalling pathway. 

## 4. Conclusions

In this research, we explored the immunotoxic effects of small-sized, highly hydrophilic SPIONs in an in vitro setting, employing a human monocytic cell line. The results revealed dose-dependent toxicity for SPIONs at doses higher than 10 μg/mL, potentially triggered by the initiation of apoptotic pathways caused by intracellular ROS generation. Surprisingly, the SPIONs did not activate two essential pathways: the Nrf2/HO-1 pathway, responsible for maintaining intracellular redox homeostasis and regulating inflammation, and the TLR-4/NF-κB pathway, which induces inflammatory cytokines. To thoroughly evaluate the safety and effectiveness of SPIONs, additional data on endocytosis mechanisms and an in-depth analysis of signal transduction pathways activation are required.

## Data Availability

Data are available on request due to restrictions.
